# AgNP-Containing Niosomes Functionalized with Fucoidan Potentiated the Intracellular Killing of *Mycobacterium abscessus* in Macrophages

**DOI:** 10.3390/ijms26031366

**Published:** 2025-02-06

**Authors:** Nereyda Niño-Martínez, Kayla Audreyartha, Kaitlyn Cheung, Sol Melchor Parra, Gabriel Martínez-Castañón, Horacio Bach

**Affiliations:** 1 Faculty of Medicine, Division of Infectious Diseases, University of British Columbia, Vancouver, BC V6H3Z6, Canada; kayla.audreyartha@gmail.com (K.A.); kcheung642@gmail.com (K.C.); 2 Facultad de Ciencias, UASLP, Av. Parque Chapultepec 1570, Privadas del Pedregal, San Luis Potosí 78295, Mexico; 3 Escuela de Ciencias, Universidad de las Americas Puebla, Puebla 72810, Mexico; sol.parramr@udlap.mx; 4 Facultad de Estomatología, Universidad Autonoma San Luis Potosí, San Luis Potosí 78290, Mexico; mtzcastanon@fciencias.uaslp.mx

**Keywords:** niosome, functionalization, fucoidan, *Mycobacterium abscessus*, silver nanoparticles, single-chain antibody, glycopeptidolipid

## Abstract

Intracellular pathogens represent a challenge for therapy because the antibiotics used need to diffuse into the cytoplasm to target the pathogens. The situation is more complicated in the mycobacteria family because members of this family infect and multiply within macrophages, the cells responsible for clearing microorganisms in the body. In addition, mycobacteria members are enclosed inside pathogen-containing vesicles or phagosomes. The treatments of these pathogens are aggravated when these pathogens acquire resistance to antibiotic molecules. As a result, new antimicrobial alternatives are needed. Niosomes are vesicles composed of cholesterol and nonionic surfactants that can be used for antibiotic encapsulation and delivery. The current study developed a systematic formulation of niosomes to determine the best option for niosome functionalizing for precise delivery to the intracellular pathogen *Mycobacterium abscessus*. Silver nanoparticles (AgNPs) were synthesized using gallic acid as an antibacterial agent. Then, niosomes were prepared and characterized, following the encapsulation of AgNPs functionalized with a single-chain antibody screened against the cell wall glycopeptidolipid of *Mycobacterium abscessus*. For a precise delivery of the cargo into macrophages, the niosomes were also functionalized with the polysaccharide fucoidan, taken specifically by the scavenger receptor class A expressed on the surface of macrophages. Results of the study showed a steady decrease in the intracellular pathogen load after 48 h post-infection. In conclusion, this system could be developed into a platform to target other types of intracellular pathogens and as an option for antimicrobial therapy.

## 1. Introduction

Pathogenic members of the Mycobacteriaceae family infect and multiply within macrophages [1]. These include *Mycobacterium tuberculosis*, *M. leprae*, *M. abscessus*, *M. avium*, and *M. ulcerans*. Because these pathogens reside and replicate within macrophages, treating them with antibiotics is challenging. To reach intracellular pathogens, drugs have to diffuse through the cell and phagosome membranes (barriers) to reach the pathogen inside the macrophage.

Besides *M. tuberculosis*, other pathogenic mycobacteria, including nontuberculous mycobacteria, have registered an infection increase worldwide. This well-reported and newly emerging problem showed that the *M. abscessus* complex increased the level of infection globally by 65% [2,3]. This pathogen is a rapidly growing pathogen causing chronic lung infection (e.g., cystic fibrosis) and skin and soft tissue infections. The treatment of this pathogen is uniquely complex and challenging due to its intrinsic antibiotic resistance [4]. Therefore, new antibiotic alternatives, like those described in this paper, are under development to combat this pathogen.

**Figure 1:**
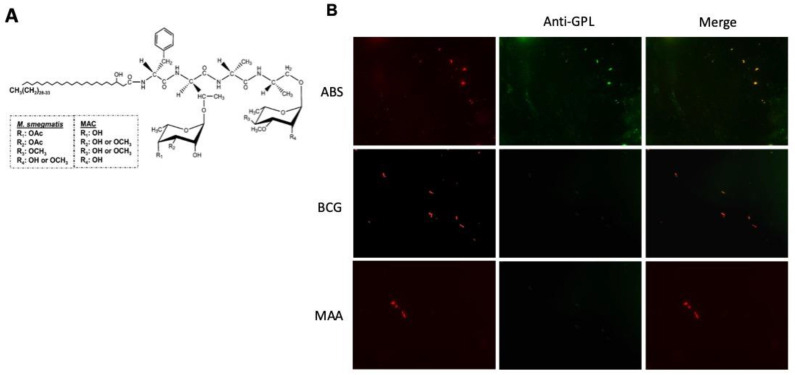
(**A**) Structure of GPL. (**B**). The antibody scFv recognizes *M. abscessus*. Bacteria were labeled with Rhodamine B (10 μg/mL, MilliporeSigma, Burlington, MA USA) and exposed to *M. abscessus,* which had previously flamed on a microscope slide. The scFv-51 (not co-expressed with the red fluorescent protein) and bacteria were incubated for 30 min, washed with PBS (×3), and incubated with FITC-mouse-anti-his tag (Thermo Fisher, Waltham, MA USA) for 30 min. The slides were analyzed by fluorescence microscopy after being washed with PBS (×3). The yellow color indicates the co-localization of scFv-51 to the pathogen. ABS, *M. abscessus*; BCG, *M. bovis* strain BCG; MAA, *M. avium avium*.

One of the cell wall components of *M. abscessus* is the glycolipopeptide (GLP, Figure 1A) [5,6]. Since this molecule is located on the outmost part of the pathogen’s cell wall, it can serve as a precise target for killing this pathogen.

One of the most popular delivery systems in medicine is the liposome. Liposomes are artificial bilayer lipid-based vesicles loaded with a particular drug. They are synthesized with a mixture of phospholipids and cholesterol [7], and the drug of interest is trapped inside them [8]. In general, this system is desirable when the drug to be delivered is toxic or hydrophobic. Therefore, once encapsulated, liposomes avoid a systemic free circulation of the drug, which can potentially cause detrimental outcomes for the body.

Over the last few years, another type of vesicle termed nonionic surfactant vesicles or niosomes has attracted the attention of researchers. This is due to advantages related to the lack of phospholipids that, in liposomes, could affect the stability, including undergoing oxidation with a concomitant degradation of the vesicles [9].

Niosomes are also artificial vesicles but differ from liposomes in their composition. The reagents used for their synthesis are surface active agents (surfactants) and cholesterol. Although niosomes have similar benefits compared to liposomes, their features still differ [10].

Several publications reported the use of molecule-loaded niosomes to target microorganisms. For example, vancomycin was shown to increase its antibacterial activity when encapsulated in niosomes against *Staphylococcus aureus* and methicillin-resistant *S. aureus* [11,12]. Other antimicrobial agents encapsulated in niosomes, such as selenium nanoparticles (SeNPs), ciprofloxacin, rifabutin, and lignin-AgNPs [13,14,15], showed antibacterial activities.

Recently, we published a study showing that niosomes encapsulated with AgNPs reduced the intracellular load of *M. abscessus* in a human-derived macrophage model of infection (THP-1) [15]. Although we showed a decrease in the survival of the intracellular pathogen in macrophages, the delivery of the niosomes was not specific to macrophages, limiting this application as a future therapy for members of the Mycobacteriaceae family. Thus, a more precise delivery system to target macrophages was developed. This developed system was more sophisticated because of the functionalization of the core and surface of the niosome for accurate delivery.

In a previous study, our team selected a human-derived single-chain antibody (scFv-51) by screening a proprietary scFv library using phage display technology against a GPL extracted from *M. smegmatis* [16]. This scFv was co-expressed with maltose-binding protein and a red fluorescent protein (scFv-51) in *E. coli* [16].

The polysaccharide fucoidan was functionalized in the current study on the niosome surface. Fucoidan is a polysaccharide obtained from seaweed known to be selectively taken by macrophages through the scavenger receptor class A [17]. In addition, we conjugated AgNPs to the scFv-51 for a precise attachment of the AgNPs to the *M. abscessus* cell wall once the niosome is fused to the macrophage membrane. The importance of fucoidan decorating the surface of the niosome results in a specific target of macrophages. The results of this study support the development of a platform to target other intracellular pathogens in macrophages by exchanging the scFv.

## 2. Results and Discussion

### 2.1. ScFv-51 Identified GPL-M. abscessus

Because the scFv-51 was screened against the GPL of *M. smegmatis*, which has slight variation compared to the *M. abscessus* complex, we exposed *M. abscessus* to scFv-51 to verify the binding. Results showed a red coloration of the bacteria using a fluorescence microscope, indicating that the red fluorescent protein (co-expressed with scFv-51) recognized the *M. abscessus* strain (Figure 1B).

### 2.2. Characterization of the Niosomes

As indicated earlier, a series of niosomes were synthesized using different ratios and mixtures. Niosomes synthesized using the surfactants Lubrol PX, Tergitol, Triton X-100, and Triton-X114 were excluded for further investigation because the parameters obtained (PDI, and the number of measured peaks) were out of the range we set up for PDI ≤ 0.3 and the number of peaks = 1. Based on the results (Table 1), representative niosomes were selected, and four niosomes, NA4, NB5, NC3, and ND3 (one per group), were chosen to continue with the functionalization.

Niosomes were synthesized using a series of surfactants and cholesterol in different ratios to find the best fit. This original screening determined that the best combination was using the nonionic surfactants Span and Tween. Other tested surfactants generated niosomes with specific parameters that were excluded from the study. This exclusion was based on the large size of the NP, high PDI value (>0.3), and number of peaks observed in DLS analysis (>1).

The Span series included the numbers 20 (monolaurate), 40 (monopalmitate), 60 (monostearate), and 80 (monooleate). The Span series comprised an ester between a fatty acid linked to a sorbitol-derived polyol (sorbitan). On the other hand, the Tween series included the same arrangement as the Span series but supplemented with a conjugated polyoxyethylene molecule (PEG) using the same numbers. Thus, the Span series is more hydrophobic compared to the Tween because of the lack of the hydrophilic portion of the PEG. The fact that combinations of the same numbers of these surfactants gave a superior quality of the niosomes is because both series have the same length of the fatty acid, differing only in the hydrophilic portion of the Tween. These differences are supported by the hydrophilic–lipophilic balance (HLB) [18], which classifies a molecule as lipophilic when the value of HBL is <10. The Span series has HBL values of 8.6, 6.7, 4.7, and 4.3 for Span 20, Span 40, Span 60, and Span 80, respectively. An HBL value > 10 defines a molecule as water soluble, and the Tween series has HBL values of 16.7, 15.6, 14.9, and 15 for Tween 20, Tween 40, Tween 60, and Tween 80, respectively [19]. In summary, the quality of the niosomes synthesized using Span–Tween combinations provides a stable balance with the Tween molecules arranged to the outmost part of the niosomes.

**Figure 2:**
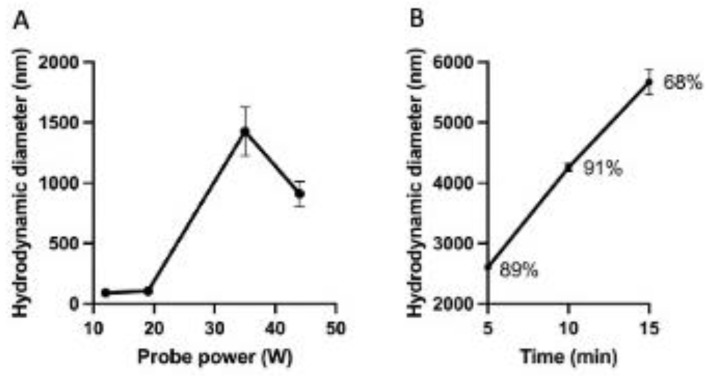
Effect of the sonication on the niosome size. NA4 niosomes were subjected to sonication during their last step of synthesis. (**A**) Impact of the sonicator power on the niosome sizes. (**B**) Impact of the sonication time on the niosome size using a probe power between 12 and 15 W. The numbers at each time point represent the highest percentage of the main peak, as measured by PDI in the DLS device.

The smallest niosome obtained was NA4, with a D_H_ of 91 nm, which was used to assess the power and the exposure time to a sonicator used in the hydration step of the niosome synthesis. Results showed that the probe power of the sonicator affected the niosome size. For example, niosome sizes of 93.9 nm and 1649 nm were measured when the average of the probe power was 13.5 W and 33 W, respectively (Figure 2A). The characterization of this series is detailed in Table 2. In addition, an increase in the niosome sizes was measured with the rise in the sonication time (Figure 2B). In conclusion, the sonication process was optimum if performed immediately after the hydration step and using a glass vial (cylindrical shape, 17 mm D × 55 mm height; 12 mL total volume capacity, placed on an ice bucket to prevent overheating during 5 min.

The sonicator parameters used in the synthesis were assessed to determine the power fitting for the smallest size of the niosomes and the sonication time. In the present study, we reported that a power ≤ of 20 W for 5 min did not change the size of the niosomes. However, there is a concomitant increase in the sizes of the niosomes with an increase in the sonication time. In addition, the main peak of the sample, as analyzed by DLS, was reduced at 15 min, indicating that a heterogeneous population is emerging. Another study encapsulating the anti-HIV drug zidovudine showed that exposure of the niosomes to sonication above 5 min caused damage to the niosomes, which reinforced our findings [20].

### 2.3. Characterization of Niosomes Encapsulated with AgNP-scFv-51 and Aminated Fucoidan

The niosomes encapsulated with AgNP-scFv-51 were characterized using DLS to measure the D_H_, PDI, ζ potential, and encapsulation efficiency. Results showed that the formulation NA4 AgNP-scFv-51 was the smallest niosomes synthesized regarding their sizes (93 nm) with an encapsulation efficiency of 43% (Table 3). However, a better encapsulation (77.6%) was achieved in NC3 AgNP-scFv-51, with a D_H_ of 120 nm.

Regarding the incorporation of fucoidan, adding 10% to NB5 AgNP-scFv-51 showed a smaller size (in nm) than 20% (Table 3). The encapsulation efficacy (EE) could not be measured because, after the disruption of the niosomes, the released AgNP-scFv-51 turned into a turbid solution.

**Figure 3:**
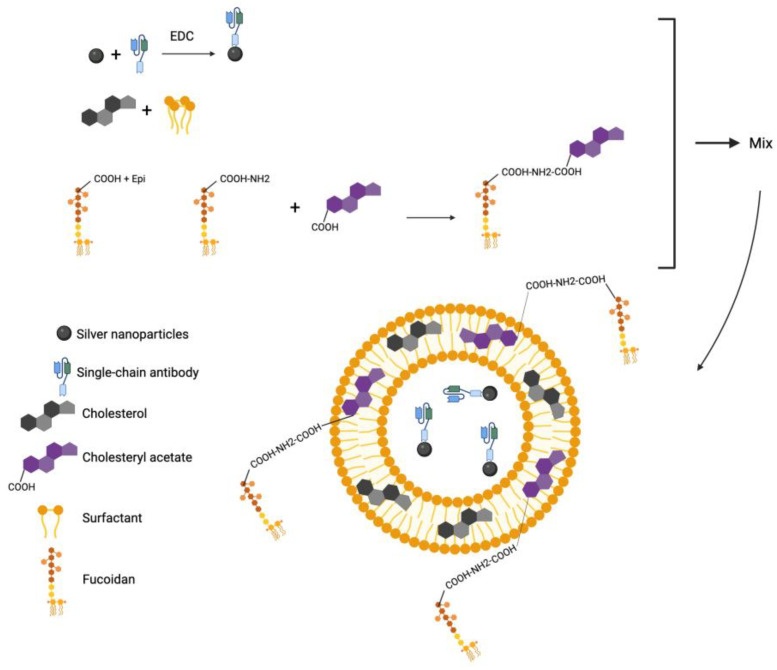
Scheme showing the synthesis of AgNP-scFv-51 with aminated fucoidan. Created with Biorender.com.

The whole process of preparing the final nanocomposite is illustrated in Figure 3.

### 2.4. Stability of Niosomes

To determine the stability of the niosomes at 4 °C, samples were assessed at the time of synthesis (Time = 0) and after two months (Time = 60 days). After 60 days, most formulations did not undergo further analysis because of their sizes or number of peaks (≥2). Only six formulations from the tested series, including NB5, NC, NC2, NC3, ND1, and ND4, showed stability (Table 4). These formulations were suspended after 60 days with no visible color changes.

Results showed that only six formulations showed satisfactory stability based on their sizes and the presence of only one peak after DLS analysis measuring PDI. Another study has also reported measurements using DLS. In that study, the PDI obtained was very high, ranging between 0.4 and 0.8. These high values indicate a heterogeneous population of niosomes (no peak number was provided) [21]. Other studies found in the literature could not be compared to ours because of the different measurements used in the niosome stability assessment. For example, reported studies used absorbance of the suspension or backscattering [22,23] or counting niosomes using Image J, which cannot assess niosomes in the nanosize range [24].

### 2.5. Cytotoxicity of the Niosomes

**Figure 4:**
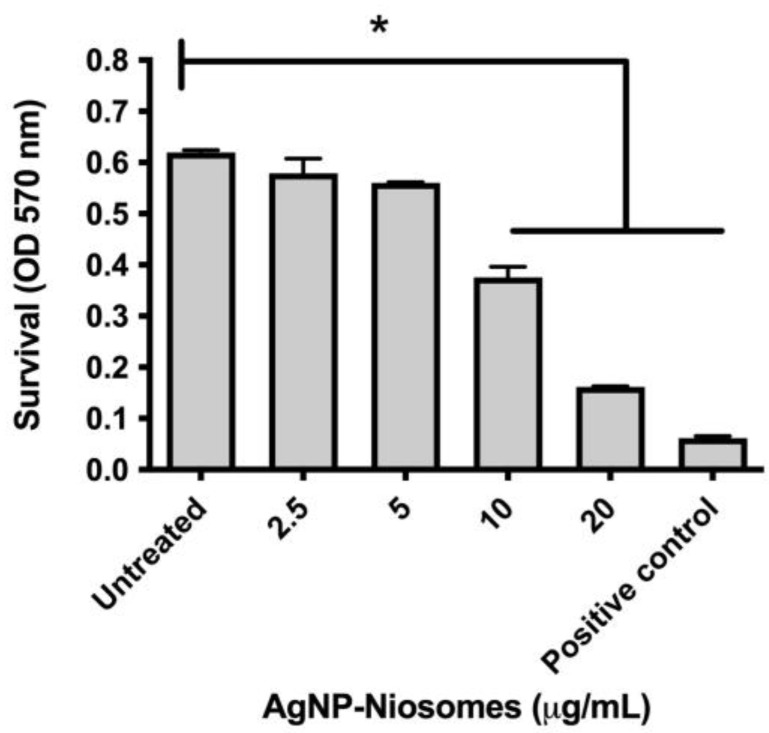
Cytotoxicity of niosomes. Differentiated THP-1 was treated with different concentrations of NC3 AgNP-scFv-51 and evaluated by MTT. Untreated and SDS-treated cells were used as negative and positive controls, respectively. OD, optical density. * *p*-value < 0.05. Experiments were performed in triplicate.

Exposure of the niosomes to THP-1 cells showed that a 5 μg/mL concentration caused no cytotoxicity to THP-1 (Figure 4), suggesting that this concentration could safely be used in the THP-1 infection with *M. abscessus*.

### 2.6. Survival of M. abscessus in Treated Niosomes

**Figure 5:**
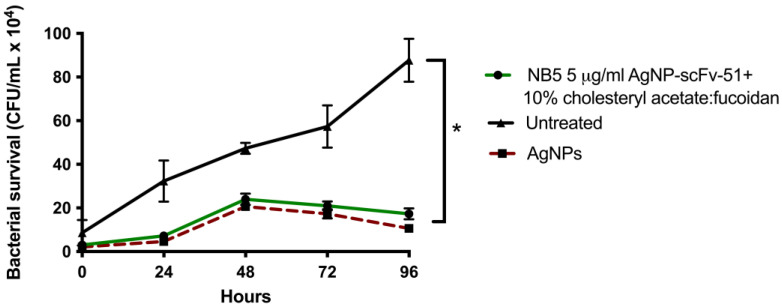
Survival of *M. abscessus* within macrophages. Differentiated THP-1 cells were infected with *M. abscessus* at a multiplicity of infection of 1. The macrophages were treated with the NB5 5 μg/mL AgNP-scFv-51 + 10% cholesteryl acetate:fucoidan. Untreated cells were used as a negative control, whereas AgNPs were used as a positive control. Shown is the median ± SD of three independent experiments. CFU, colony-forming units. * *p*-value <0.05.

The survival of *M. abscessus* in treated macrophages with AgNP:scFv-5:fucoidan-10% showed a significant decrease in the CFU/mL counting starting at 48 h, with a concomitant decrease observed at 72 and 96 h post-infection (Figure 5).

The encapsulation of the AgNP:scFv-51 showed niosome sizes ranging between 93 and 864 nm with PDIs < 30% and a maximal EE of 77.6%. Other studies reported higher EE but with different niosome parameters regarding their sizes and PDIs, which might have limited applications. For example, a study reported sizes ranging between 195 and 893 nm with PDIs varying between 0.381 and 0.725 when the antibiotic rifampicin was encapsulated [25]. Although a similar size range was obtained in our study, our AgNP:scFv-51 size was more significant than the rifampicin size (823 Da). In addition, the PDIs of all of our formulations were superior (<0.30) compared to the other study (0.381–0.725). A PDI > 0.30 indicates that the particle or vesicle undergoes aggregation with a potential to precipitate, suggesting that our formulations will be more stable. Another study reported the synthesis of niosome encapsulation of the drug zidovudine with sizes of 279 and 282 nm when Span 20 and Tween 80 were used as surfactants, respectively. PDIs of 0.43 and 0.49 were also measured in this case, respectively [20]. Our study reported better sizes and PDIs than other studies using only a single surfactant.

Since members of the pathogenic mycobacteria infect and multiply within macrophages, a precise delivery to these cells will provide benefits compared to a systemic administration of drugs. With this in mind, we prepared niosomes containing fucoidan, adding the polysaccharide fucoidan. Macrophages have a high affinity for fucoidan, a polysaccharide ligand of the scavenger receptor class A, and a high affinity to macrophages [17]. This scavenger receptor class A is expressed on the surface of macrophages and dendritic cells [26]. This suggests that the niosomes containing fucoidan:AgNP:scFv-51 could be precisely delivered to macrophages with limited or no binding to other human cells.

To verify whether fucoidan:AgNP:scFv-51 could induce a cytotoxic effect on macrophages, THP-1 cells were exposed to the fucoidan:AgNP:scFv-51, and the cytotoxicity was assessed by MTT. Results showed that a 5 mg/mL concentration caused no cytotoxic effect on the macrophages. This concentration was used in the *M. abscessus* survival upon macrophage infection. After treating macrophages infected with *M. abscessus* with these niosomes mentioned above, results showed that supplementing the niosome containing fucoidan:AgNP:scFv-51 gradually reduced the pathogen’s survival. This significant decrease could be observed after 48 h post-treatment, extending through the end of the experiment at 96 h. In a previous study from our lab, we used niosomes encapsulated with AgNPs only and used the same infection model of *M. abscessus*-infected macrophages. In that report, a sharp decrease in the pathogen’s survival was observed from the beginning of the experiment [15]. Since the entrance of the AgNPs was based on the fusion between the niosome and macrophage membranes, direct delivery of the AgNPs inside the cytoplasm could directly attack and reduce the number of *M. abscessus*. In our current experiment, this reduction in the number of the pathogen might indicate that the entrance of the fucoidan:AgNP:scFv-51-containing niosome proceeded through the scavenger receptor class A and not as a fusion process. However, this statement should be verified in future works. Thus, the entrance of the fucoidan:AgNP:scFv-51 into the cytoplasm occurred with a delay compared to the niosomes loaded with only AgNPs.

Our study cannot be compared to the literature because, to our knowledge, this is the first synthesis of functionalized niosomes for a precise target of intracellular pathogens.

Other studies have shown the benefits of antibiotics encapsulated in niosomes. For example, ciprofloxacin niosomes exhibit significant antibacterial activity against *S. aureus* and *Escherichia coli* [27]. Although significant results were also obtained for the pathogen *F. tularensis*, the delivery system was liposomes, not niosomes [28]. Another study functionalized the surface of niosomes with methoxy-polyethylene glycol and oleylamine. Results showed a superior activity against *S. aureus* and MRSA using vancomycin as the encapsulated antibiotic [11].

Other studies evaluated the use of SeNPs encapsulated in niosomes in planktonic pathogens. Results showed a potent antimicrobial activity against *S. aureus*, *Enterococcus faecalis*, *E. coli*, and *Pseudomonas aeruginosa* [14]. Although the results were encouraging, the antibacterial activity was not shown in infected macrophages.

In conclusion, we developed a new functionalized niosome with increasing complexity by including new functionalization. The best fit of the synthesized niosomes was selected for further experiment to encapsulate AgNPs conjugated to the antibody scFv-51 specific to the cell wall of *M. abscessus*. We concluded that the complex functionalization described in this study could control the multiplication of intracellular *M. abscessus*. This functionalization could increase the proximity of the AgNPs to the pathogen inside the cytoplasm and result in the adsorption of the AgNPs to the pathogen cell wall. As a result of having the AgNPs adhered to the pathogen, a potential generation of reactive oxygen species in situ could increase the precision of the pathogen killing. The knowledge obtained from this study could be developed to treat other intracellular pathogens (e.g., *M. tuberculosis*, *Listeria monocytogenes*, *F. tularensis*, *Legionella pneumophila*, etc.) infecting macrophages or other cells.

## 3. Materials and Methods

### 3.1. ScFv-51 Validation Against GPL-M. abscessus

The scFv-51 was subcloned into pMAL and purified using an amylose resin (NEB, Ipswhich, MA, USA) according to the manufacturer’s instructions. The purified scFv-51 was assayed against an *M. abscessus* GPL-producer strain (kindly provided by Dr. Jean-Louis Herrmann, University Versailles-Saint-Quentin, France). The strain was cultured in 7H9 broth (B&D, Franklin Lakes, NJ, USA) supplemented with 10% OADC (B&D) and 0.05% Tween-80. The strain was statically cultured in an incubator at 37 °C for 3 days, washed with PBS (× 3), deionized water (× 3), and 10 μL was deposited on a microscope slide. The drop was spread over the surface of the slide and flamed to adhere to the bacteria, followed by exposure to 20 μg of scFv-51 in 100 μL PBS for 30 min. Next, the cells were washed with PBS (× 3) for 5 min each and then analyzed in an epifluorescence microscope (Zeiss Axioplan II; Carl Zeiss Inc., Thornwood, NY, USA).

### 3.2. Synthesis and Characterization of AgNPs

AgNPs were prepared using an aqueous chemical reduction, as published [29]. Briefly, 45 mL of deionized water containing 85 mg of AgNO_3_ was mixed under magnetic stirring and at room temperature with 50 mg of gallic acid (MilliporeSigma) previously dissolved in 5 mL of deionized water. Immediately after, the pH of this mixture was adjusted to 11.0 using NaOH (3.0 M). Finally, AgNPs were purified using sterile water and a P10 (GE, Chicago, IL, USA) column to separate individual components not integrated into the niosome. The volume obtained with the AgNPs was dried out in a 60 °C incubator, and the final weight of the AgNPs was determined by weight difference before and after the drying process. The AgNPs were then stored in dark conditions at room temperature until further use.

The AgNPs were characterized according to their size (D_H_), PDI, and zeta potential using dynamic light scattering (Litesizer DLS 500, Anton Paar, Graz, Austria).

### 3.3. Conjugation of AgNPs and scFv-51

The conjugation of the AgNPs to scFv-51 was performed by activating the NP surface using 1-ethyl-3-(-3-dimethylaminopropyl) carbodiimide hydrochloride (EDC, Pierce, Appleton, WI, USA) and according to the manufacturer’s instructions. Briefly, scFv-51 was dialyzed in 1 L 0.1 M MES buffer (pH 5.5) for 8 h. Then, 10 mg of EDC was dissolved in 1 mL of sterile deionized water, and 100 μL of the solution was immediately mixed with a mixture containing scFv-51 and AgNPs (mass ratio 2.75:1 AgNP:scFv-51). The mixture was left to react for 30 min at room temperature. The reaction was quenched using hydroxylamine (10 mM), and the AgNP:scFv-51 conjugates were purified using a P10 column and endotoxin-free water and stored at 4 °C.

### 3.4. Synthesis and Characterization of Niosomes

Niosomes were prepared using the thin-film hydration technique [30]. Briefly, varying amounts of surfactants and a fixed amount of cholesterol (40 mg) were dissolved in 10 mL of chloroform. The following nonionic surfactants (from MilliporeSigma or Fisher, Pittsburgh, PA, USA) were assessed: Brij 58, Lubrol PX, Span-20, Span-40, Span-60, Span-80, Tergitol NP7, Triton X-100, Triton X-114, Tween-20, Tween-40, Tween-60, and Tween-80.

The surfactant pairs used were Span-20/Tween-20, Span-40/Tween-40, Span-60/Tween-60, and Span-80/Tween-80. Different ratios of surfactants and cholesterol were assessed, as indicated in Table 1. Then, the mixture was vaporized in a rotary evaporator (Buchi, Flawil, Switzerland) for 50 min at a reduced pressure, 100 rpm, and 50 °C. Afterward, 10 mL of deionized water was added to hydrate the film in a rotary evaporator for 60 min (35 °C and 100 rpm). After that, the solution was sonicated with a tip sonicator (Misonix 3000, Farmingdale, NY, USA) for 5 min to obtain niosomes, followed by niosomes purification using a P10 column to separate the components not included within the niosomes. Samples were stored at 4 °C. Niosomes were characterized using DLS as mentioned above.

The formulation NB4 with a size of 91 nm (Table 1) was chosen to evaluate the effect of the sonication power on the sizes of the niosomes. The power of the sonicator probe (Misonix) was assessed between 10 and 40 W, with sonication times of 5, 10, and 15 min (Table 2).

### 3.5. Functionalization of Fucoidan and Conjugation with Cholesterol

An amination modification was performed on the fucoidan to generate aminated fucoidan, which was conjugated with cholesteryl acetate using EDC. The conjugation was carried out by mixing 500 mg of fucoidan (from *Macrocystis pyrifera*, MilliporeSigma), 0.65 mmol of NaOH, and 1.88 mmol of epichlorohydrin (MilliporeSigma) dissolved in 2.3 mL of deionized water. This mixture was stirred at 40 °C for 2 h. Then, it was dialyzed against deionized water supplemented with 10 mM Tris-HCl, pH 7.0, using a dialysis bag (cut-off 10,000 Da, Fisher) for 48 h at 4 °C with a water exchange at 24 h. Then, the product was dried out using a Centrivap concentrator (Labconco, Kansas City, MO, USA). When the sample was dried, the pellet was resuspended in 3 mL of aqueous ammonia (30%, VWR, Radnor, PA, USA) and stirred at 40 °C for 2 h using a water bath. After this time, the aminated fucoidan was dialyzed again as described above for 24 h and stored at 4 °C.

At this stage, the aminated fucoidan was ready for conjugation with cholesterol. Since cholesterol does not have a carboxylic group, we chose cholesteryl acetate (MilliporeSigma) to facilitate the conjugation with the aminated fucoidan using EDC and according to the manufacturer’s instructions. Briefly, 4.4 mg of cholesteryl acetate was dissolved in 1 mL of a 50% ethanol–water solution, then a mixture of EDC/NHS (molar ratio 1:1:2 with cholesteryl acetate) was added. After that, 1.275 mg of aminated fucoidan was added and incubated for 30 min at room temperature. The sample was stored at 4 °C.

To verify the conjugation between aminated fucoidan and cholesteryl acetate, a TLC analysis was performed using a glass coated with silica gel 60 F_254_ (Merck Rahway, NJ, USA) and a solvent mixture of 8 mL hexane, 2 mL ethyl ether, and 0.15 mL acetic acid. Results showed that the cholesteryl acetate migrated to a different position than the cholesteryl acetate conjugated to fucoidan (Figure S2). The TLC plate was dried and developed by charring after soaking in a solution of vanillin (10%) in H_2_SO_4_ (Figure S2).

Results of the TLC confirmed that the aminated fucoidan conjugated to the cholesteryl acetate because the conjugate could not migrate from the spotting line with the solvent because of the high polarity compared to the cholesteryl acetate, which migrated with the solvents from the spotting line.

### 3.6. Synthesis of Niosomes Using Cholesteryl Acetate

After screening the surfactants used to prepare niosomes (Table 1), we selected niosome NB5, which contained Span-60/Tween-60/cholesterol (0.2:0.3:1) (Table S1). This selection was based on assessing the lowest values of the hydrodynamic diameter (D_H_), the polydispersity index (PDI), and the presence of one peak (Figure S1). Two concentrations (10% and 20%) were tested to evaluate the effect of cholesteryl acetate on niosome synthesis.

### 3.7. Synthesis of the Functionalized Niosome

The final version of the functionalized niosome AgNP-scFv-51 with fucoidan was prepared by replacing the cholesterol in the original formulation with cholesteryl acetate conjugated to fucoidan following the same procedure detailed in the synthesis of niosomes. The amounts of the cholesterol and the surfactants remained the same. Finally, the hydration step using deionized water contained 0, 50, or 100 μg/mL of the conjugate AgNP-scFv-51, generating the final formulations defined as CXN, CXN50, and CXN100.

### 3.8. Encapsulation Efficiency of the AgNP-scFv-51 and the Complex AgNP-scFv-51 with Aminated Fucoidan

As mentioned above, four formulations of niosomes (NA4, NB5, NC3, and ND3, Table 1) were selected from all of the variations synthesized to encapsulate AgNP-scFv-51 and to generate NA4:Ag:scFv-51, NB5:Ag:scFv-51, NC3:Ag:scFv-51, and ND3:Ag:scFv-51.

To calculate the encapsulation efficiency, 1 mL of each formulation of AgNPs was centrifuged at 14,500 rpm for 10 min, and the released AgNPs concentration in the supernatant was calculated using the absorbance value of the surface plasmon of the NP at 403.8 nm. A calibration curve was plotted to determine the final concentration of the AgNPs in the solution. The encapsulation efficiency (EE) was calculated using the following formula:

$$EE\left(\%\right)=\frac{initial\;concentration\;of\;AgNPs-concentration\;of\;AgNPs\;in\;supernatant}{initial\;concentration\;of\;AgNPS}\times 100$$

In the case of the synthesis of the final complex AgNP-scFv-51 with aminated fucoidan, a similar procedure was followed for the synthesis of the niosomes. Briefly, cholesterol and the detergent (see Table 3 for ratios) were dissolved together, and after cholesterol volatilization, cholesteryl acetate conjugated to aminated fucoidan and AgNP-scFv-51 were added in the hydration step. After 1 h, the mixture was sonicated.

### 3.9. Cytotoxicity of Niosomes

The cytotoxicity of the niosomes was assessed on the human-derived monocytes THP-1 (ATCC TIB-202) and according to a previous publication [15]. 3-(4,5-dimethylthiazol-2-yl)-2,5-diphenyltetrazolium bromide (MTT) was used to measure the cytotoxic effects of the formulations. Experiments were performed in triplicate. Untreated and 1% SDS-treated cells were used as negative and positive controls, respectively.

### 3.10. Infection of Macrophages

Differentiated macrophages were infected with *M. abscessus* at a multiplicity of infection of 1. The infections were performed according to a previous publication from our lab [15]. Briefly, THP-1 monocytes were differentiated into macrophages with 20 ng/mL phorbol-myristate-acetate (Sigma, Tokyo, Japan). Sterile 24-well microplates were dispensed with 1 × 10^5^ cells/well and cultured in an incubator at 37 °C supplemented with 5% CO_2_. The next day, *M. abscessus* was opsonized with human serum for 30 min and added to each well. After 4 h, non-internalized bacteria were carefully removed by washing with PBS. Then, 5 μL of the formulation NB5:cholesteryl acetate_10/90_ was added to the infected macrophages and cultured for 96 h. Samples were sampled every 24 h, serially diluted, and plated on a solidified 7H9 media (B&D) supplemented with 10% OADC (B&D). The plates were sealed with parafilm and incubated at 37 °C for four days. Then, the colonies were counted and expressed as colony-forming units (CFU)/mL. Experiments were performed in triplicate.

## Figures and Tables

**Table 1:** Characterization of the niosomes using DLS.

Surfactant	Sample	Molar Ratio (Span:Tween:Chol)	D_H_ ± SD (nm)	PDI (%)	ζ Potential (mV)
Span-80 + Tween-80	NA1	3:0:1	712 ± 79.6	28.72	−38.8
	NA2	2.4:0.6:1	340 ± 168	26.43	−17.9
	NA3	1.8:1.2:1	367 ± 534	27.13	−10.2
	NA4	1.5:1.5:1	91 ± 79.9	25.14	−5.80
	NA5	1.2:1.8:1	245 ± 218	25.26	−1.11
	NA6	0.6:2.4:1	504 ± 315	27.47	−0.26
	NA7	0:3:1	200 ± 151	29.29	+0.32
Span-60 + Tween-60	NB1	0.5:0:1	251 ± 75	14.17	−23.1
	NB2	0.4:0.1:1	289 ± 155	25.05	−25.4
	NB3	0.3:0.2:1	429 ± 349	23.46	−20.7
	NB4	0.25:0.25:1	262 ± 156	24.70	−21.2
	NB5	0.2:0.3:1	200 ± 115	19.44	−26.4
	NB6	0.1:0.4:1	276 ± 137	24.42	−12.3
	NB7	0:0.5:1	120 ± 102	24.93	−23.3
Span-40 + Tween-40	NC1	2:0:1	181 ± 65	17.79	−33.9
	NC2	1.6:0.4:1	740 ± 23	35.12	−39.5
	NC3	1.2:0.8:1	134 ± 13	28.52	−13.2
	NC4	1:1:1	275 ± 189	33.07	−27.9
	NC5	0.8:1.2:1	120 ± 76	27.84	−9.74
	NC6	0.4:1.6:1	354 ± 91	38.31	−8.37
	NC7	0:2:1	17 ± 4	25.98	−10.0
Span-20 + Tween-20	ND1	1:0:1	170 ± 54	20.84	−44.3
	ND2	0.8:0.2:1	246 ± 80	24.28	−25.3
	ND3	0.6:0.4:1	170 ± 287	26.52	−17.1
	ND4	0.5:0.5:1	224 ± 228	30.67	−26.0
	ND5	0.4:0.6:1	244 ± 81	29.13	−18.1
	ND6	0.2:0.8:1	333 ± 287	25.75	−19.7
	ND7	0:1:1	430 ± 228	27.71	+5.72

Chol, cholesterol; D_H_, hydrodynamic diameter; PDI, polydispersity index; ζ, zeta.

**Table 2:** Characterization of niosomes loaded with 0, 50, or 100 μg/mL of the conjugate AgNP-scFv-51 using DLS.

Sample	Molar Ratio (Span:Tween:Chol)	D_H_ ± SD (nm)	PDI (%)	ζ Potential (mV)
CXN	0.2:0.3:1	195 ± 64	26.99	−11.5
CXN50	0.2:0.3:1	470 ± 366	26.19	−12.2
CXN100	0.2:0.3:1	149 ± 137	30.87	−7.85

D_H_, hydrodynamic diameter; PDI, polydispersity index; ζ, zeta.

**Table 3:** Characterization of AgNP-scFv-51-loaded niosomes.

Sample	Molar Ratio (Span:Tween:Chol)	D_H_ ± SD (nm)	PDI (%)	ζ Potential (mV)	EE (%)
Ag	N/A	23.4 ± 1.8	23.13	−42.8	N/A
AgNP-scFv-51	N/A	4406 ± 1046	5.58	−6.62	N/A
NA4 AgNP-scFv-51	1.5:1.5:1	93 ± 91	28.31	−1.06	43.5
NB5 AgNP-scFv-51	0.2:0.3:1	309 ± 342	28.14	−1.24	71.3
NC3 AgNP-scFv-51	1.2:0.8:1	120 ± 365	25.87	−2.42	77.6
ND3 AgNP-scFv-51	0.6:0.4:1	864 ± 589	26.60	−12.7	63.0
NB5 AgNP-scFv-51	0.2:0.3:1	372 ± 249	28.95	−1.14	NM
NB5 AgNP-scFv-51 + 20% CA:fucoidan	0.2:0.3:1	244 ± 83	27.12	+0.65	NM
NB5 AgNP-scFv-51 + 10% CA:fucoidan	0.2:0.3:1	149 ± 86	26.74	−1.32	NM

CA, cholesteryl acetate; D_H_, hydrodynamic diameter; NM, not measured; PDI, polydispersity index; ζ, zeta.

**Table 4:** Stability of niosomes by DLS after 60 days.

Series	Time = 0	Time = 60 Days
**NA**		
*NA1*		
D_H_ (nm)	712.9	823
PDI (%)	28.72	28.93
Peak analysis intensity *	1, 551.6 (100%)	2, 1329 (74.71%); 188.2 (29.29%)
ζ potential (mV)	−38.85	−26.59
*NA2*		
D_H_ (nm)	340.8	315.1
PDI (%)	26.43	21.76
Peak analysis intensity *	2, 391.7 (94.03%); 43.9 (5.97%)	1, 344.8 (100%)
ζ potential (mV)	−17.96	−20.49
*NA3*		
D_H_ (nm)	367.3	241.3
PDI (%)	27.13	19.87
Peak analysis intensity *	2, 466.4 (98.23%); 21.85 (1.77%)	2, 252.7 (96.87%); 15.05 (3.13%)
ζ potential (mV)	−10.21	−7.45
*NA4*		
D_H_ (nm)	91.7	265.4
PDI (%)	25.14	30.08
Peak analysis intensity *	1, 112.8 (100%)	2, 307.1 (90.75%); 36.7 (9.25%)
ζ potential (mV)	−5.8	−5.37
*NA5*		
D_H_ (nm)	245.7	192.4
PDI (%)	25.26	30.86
Peak analysis intensity *	2, 282.7 (95.73%); 22.05 (4.27%)	2, 241.4 (92.34%); 24.23 (7.66%)
ζ potential (mV)	−1.11	−0.23
*NA6*		
D_H_ (nm)	504.5	280.5
PDI (%)	27.47	29
Peak analysis intensity *	3, 804.3 (66.66%); 137.7 (29.09%);	2, 367.9, (89.71%); 27.3 (10.29%)
ζ potential (mV)	−0.26	−0.27
*NA7*		
D_H_ (nm)	200.7	111.7
PDI (%)	29.29	29.44
Peak analysis intensity *	2, 317.3 (79.62%); 29.63 (20.38%)	2, 188.0 (78.6%); 26.51 (21.35%)
ζ potential (mV)	+0.32 mV	−0.31
**NB**		
*NB1*		
D_H_ (nm)	251.5	247.2
PDI (%)	14.17	14.05
Peak analysis intensity *	1, 255.8 (100%)	1, 223.9 (100%)
ζ potential (mV)	−23.1	−19.9
*NB2*		
D_H_ (nm)	289.8	286.8
PDI (%)	25.05	19.16
Peak analysis intensity *	1, 310.6, (100%)	1, 321.9, (100%)
ζ potential (mV)	−25.46	−26.4
*NB3*		
D_H_ (nm)	429.5	446.9
PDI (%)	23.46	30.03
Peak analysis intensity *	2, 799.3 (52.31%); 173.6 (49.69%)	2, 555.1 (85.69%); 81.8 (14.31%)
ζ potential (mV)	−20.77	−34.6
*NB4*		
D_H_ (nm)	262.5	351.8
PDI (%)	24.7	24.54
Peak analysis intensity *	1, 392.4 (100%)	2, 714.5 (50.51%); 168.9 (49.5%)
ζ potential (mV)	−21.2	−32.5
*NB5*		
D_H_ (nm)	200.8	185.9
PDI (%)	19.44	23.43
Peak analysis intensity *	1, 244.1 (100%)	1, 223.2 (100%)
ζ potential (mV)	−26.4	−27.4
*NB6*		
D D_H_ (nm)	276.7	261.3
PDI (%)	24.42	26.61
Peak analysis intensity *	2, 305.5 (98.10%); 29.46 (1.90%)	1, 304.6 (100%)
ζ potential (mV)	−12.36	−23
*NB7*		
D_H_ (nm)	120.1	107.4
PDI (%)	24.93	23.54
Peak analysis intensity *	2, 140.7 (94.93%); 13.94 (5.07%)	2, 129.3 (96.88%); 14.15 (3.12%)
ζ potential (mV)	−23.37	−13.2
**NC**		
*NC1*		
D_H_ (nm)	181	166.6
PDI (%)	17.79	14.14
Peak analysis intensity *	1, 186.9 (100%)	1, 162.1 (100%)
ζ potential (mV)	−33.9	−38.5
*NC2*		
D_H_ (nm)	740.9	225.7
PDI (%)	35.12	23.51
Peak analysis intensity *	2, 919.4 (83.01%); 121.2 (16.99%)	1, 222.3 (100%)
ζ potential (mV)	−39.53	−41.45
*NC3*		
D_H_ (nm)	134.9	136.2
PDI (%)	28.52	28.63
Peak analysis intensity *	1, 211.4 (100%)	1, 185.1 (100%)
ζ potential (mV)	−13.2	−9.69
*NC4*		
D_H_ (nm)	275	251.7
PDI (%)	33.07	33.85
Peak analysis intensity *	2, 654.6 (61.61%); 57.13 (35.19%);	3, 545.3 (63.87%); 55.66 (33.23 %); 10.93 (2.89%)
ζ potential (mV)	−27.9	
*NC5*		
D_H_ (nm)	120.2	103.2
PDI (%)	27.84	28.52
Peak analysis intensity *	2, 95.16 (73.91%); 621.2 (26.09%)	2, 117 (88.99%); 1033 (11.01%)
ζ potential (mV)	−9.74	−9.74
*NC6*		
D_H_ (nm)	354.5	48.58
PDI (%)	38.31	32.64
Peak analysis intensity *	3, 1303 (46.40%); 560.4 (28.73%);	3, 499.2 (32.53%); 5402 (28.46%); 24.9 (39.02%)
ζ potential (mV)	−8.37	
*NC7*		
D_H_ (nm)	17.19	15.09
PDI (%)	25.98	23.55
Peak analysis intensity *	2, 13.7 (76.90%); 727.2 (23.10%)	3, 13.2 (77.78%); 2166 (14.04%);
ζ potential (mV)	−10.02	−10.02
**ND**		
*ND1*		
D_H_ (nm)	170.4	197.7
PDI (%)	20.84	13.22
Peak analysis intensity *	1, 178.3 (100 %)	1, 202.5 (100%)
ζ potential (mV)	−44.38	−35.17
*ND2*		
D_H_ (nm)	246.9	175
PDI (%)	24.28	26.93
Peak analysis intensity *	2, 263.8 (95.13%); 29.04 (4.87%)	2, 223.5 (97.36%); 21.9 (2.64%)
ζ potential (mV)	−25.31	−23.03
*ND3*		
D_H_ (nm)	170.1	212.2
PDI (%)	26.52	29.27
Peak analysis intensity *	2, 218.0 (92.48%); 36.55 (7.52%)	2, 262.0 (88.80%); 34.1 (11.20%)
ζ potential (mV)	−17.13	−18.63
*ND4*		
D_H_ (nm)	224.8	127
PDI (%)	30.67	26.82
Peak analysis intensity *	3, 249.8 (84.58%); 1455 (14.42%);	1, 157.2 (100%)
ζ potential (mV)	−26	−25.38
*ND5*		
D_H_ (nm)	244.5	136.1
PDI (%)	29.13	27.25
Peak analysis intensity *	2, 270.3 (92.22 %); 33.65 (7.78%)	2, 179.6 (91.78%); 26.1 (8.22%)
ζ potential (mV)	−18.15	−20.74
*ND6*		
D_H_ (nm)	333.8	195.1
PDI (%)	25.75	24.83
Peak analysis intensity *	3, 824.5 (42.67%); 138.8 (55.78%);	2, 251.7 (95.48%); 20.0 (4.52%)
ζ potential (mV)	−19.78	−25.5
*ND7*		
D_H_ (nm)	430.9	502.7
PDI (%)	27.71	31.78
Peak analysis intensity *	2, 496.8 (93.65%); 67.0 (6.35%)	3, 1121 (66.21%); 181.4 (31.14%); 23.11 (2.65%)
ζ potential (mV)	5.72	

* Number of peaks.

## Data Availability

Data will be made available on request.

## Supplementary Materials

The following supporting information can be downloaded at https://www.mdpi.com/article/10.3390/ijms26031366/s1.
