# Supplementary material for: AgNP-Containing Niosomes Functionalized with Fucoidan Potentiated the Intracellular Killing of Mycobacterium abscessus in Macrophages
Source: Int J Mol Sci. 2025 Feb 6;26(3):1366. doi: 10.3390/ijms26031366 (PMC11818696; doi:10.3390/ijms26031366)
Supplement: Supplementary file 1 [file ijms-26-01366-s001.zip › ijms-3417443-supplementary Figure S1 and Table S1.pdf]

Niosomas NA Span80:Tween 80

NA1
Hydrodynamic diameter
712.9 nm
PDI: 28.72 %
Peak analysis intensity
551.6 nm 100%
Zeta potential: -38.85mV

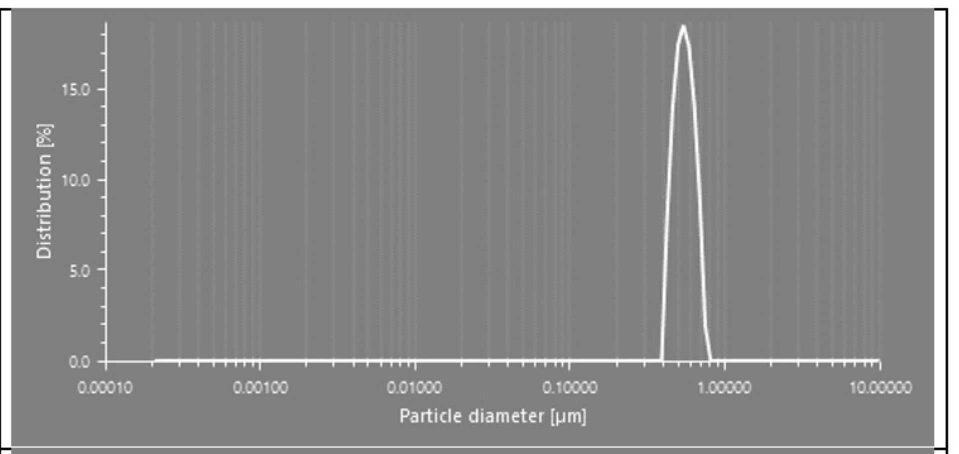

NA2
Hydrodynamic diameter
340.8 nm
PDI: 26.43 %
Peak analysis intensity
391.7 nm 94.03%
43.9 nm 5.97%
Zeta potential: -17.96mV

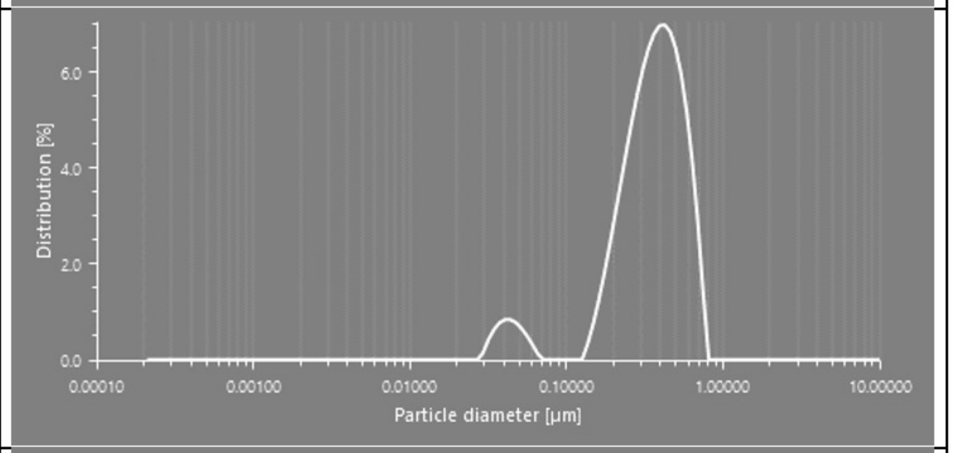

NA3
Hydrodynamic diameter
367.3 nm
PDI: 27.13 %
Peak analysis intensity
466.4 nm 98.23%
21.85 nm 1.77%
Zeta potential: -10.21mV

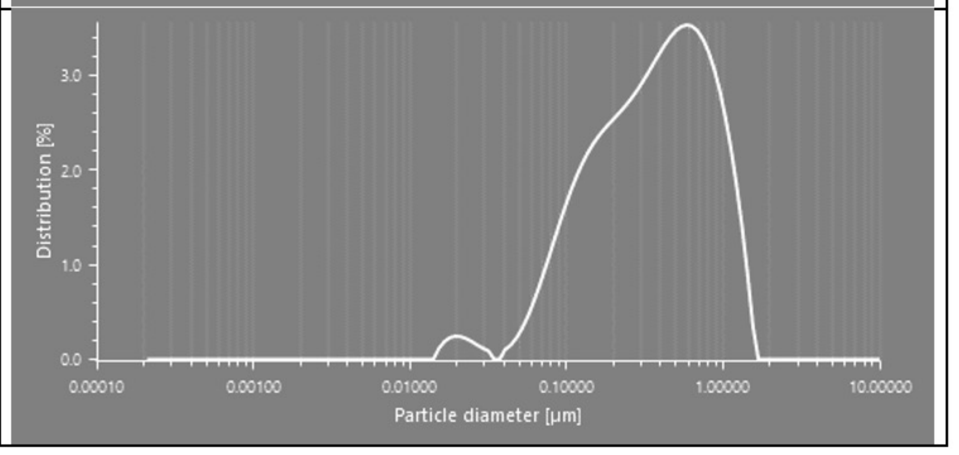

NA 50s80 50t80 2
NA4 purif
Hydrodynamic diameter
91.7 nm
PDI: 25.14 %
Peak analysis intensity
112.8 nm 100%
Zeta potential: -5.80 mV

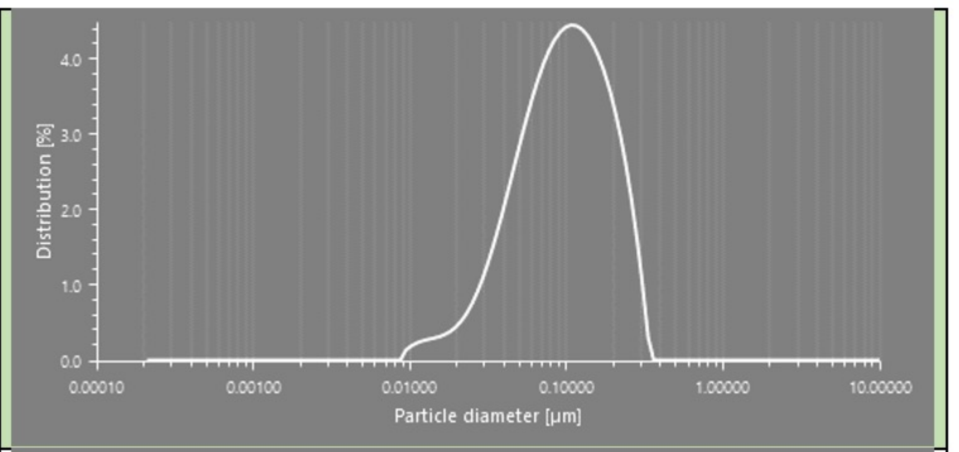

NA5
Hydrodynamic diameter
245.7 nm
PDI: 25.26 %
Peak analysis intensity
282.7 nm 95.73%
22.05 nm 4.27%
Zeta potential: -1.11 mV

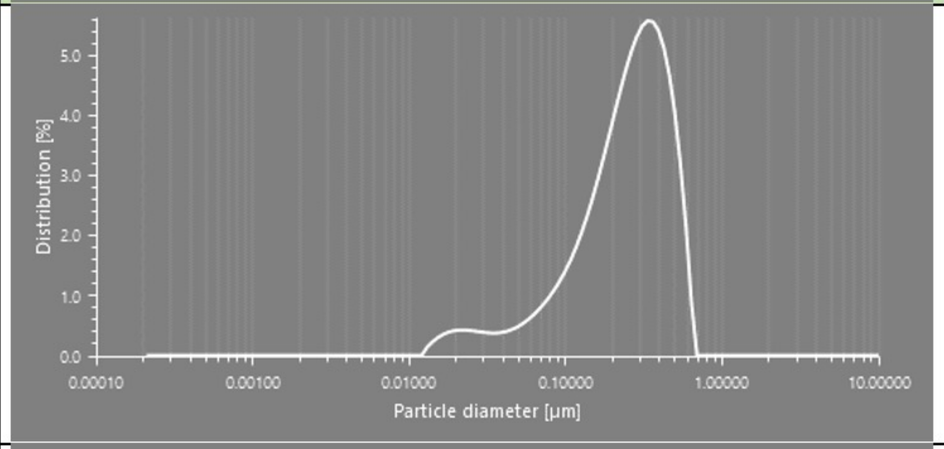

NA6
Hydrodynamic diameter
504.5 nm
PDI: 27.47 %
Peak analysis intensity
804.3 nm 66.66%
137.7 nm 29.09%
17.61 nm 4.25%
Zeta potential: -0.26 mV

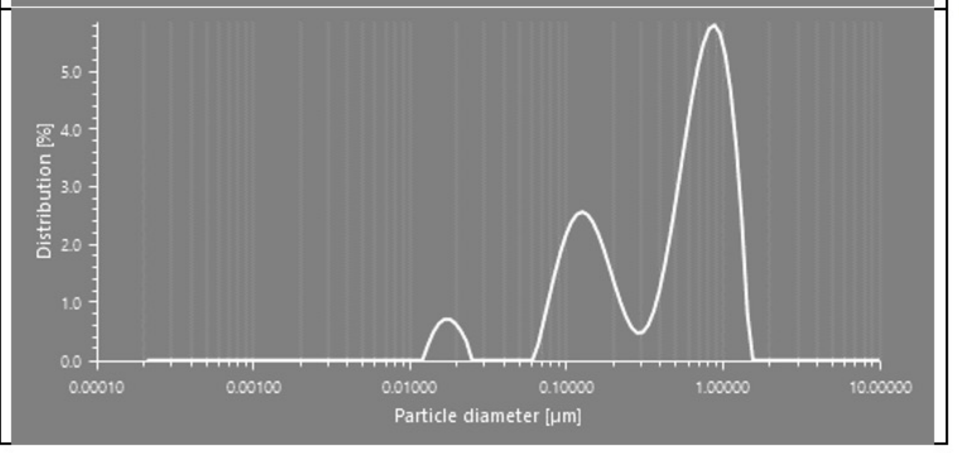

NA7
Hydrodynamic diameter
200.7 nm
PDI: 29.29 %
Peak analysis intensity
317.3 nm 79.62%
29.63 nm 20.38%
Zeta potential: +0.32 mV

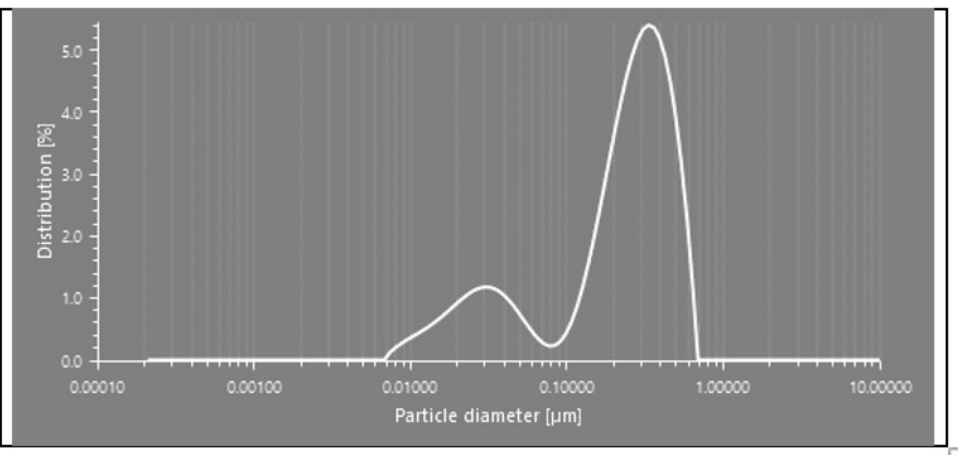

Niosomas NB Span60:Tween 60

NB1
Hydrodynamic diameter
251.5 nm
PDI: 14.17 %
Peak analysis intensity
255.8 nm 100%
Zeta potential: - 23.10 mV

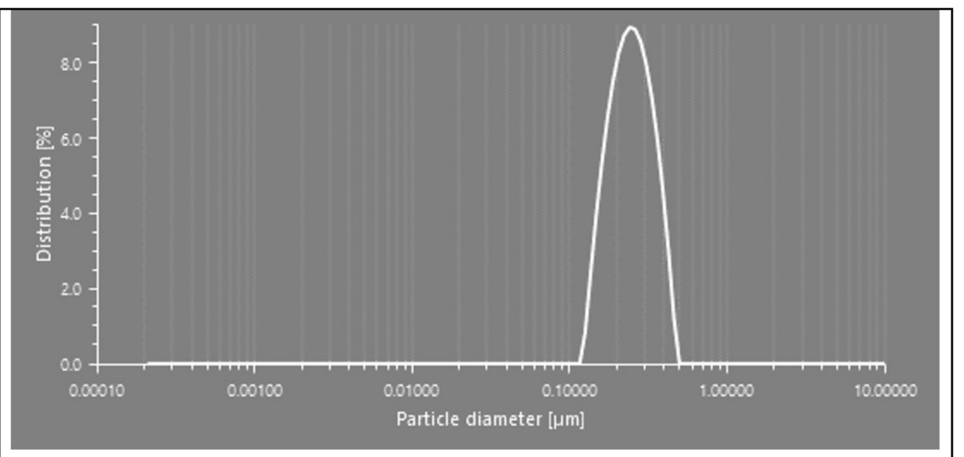

NB2
Hydrodynamic diameter
289.8 nm
PDI: 25.05 %
Peak analysis intensity
310.6 nm 100%
Zeta potential: - 25.46 mV

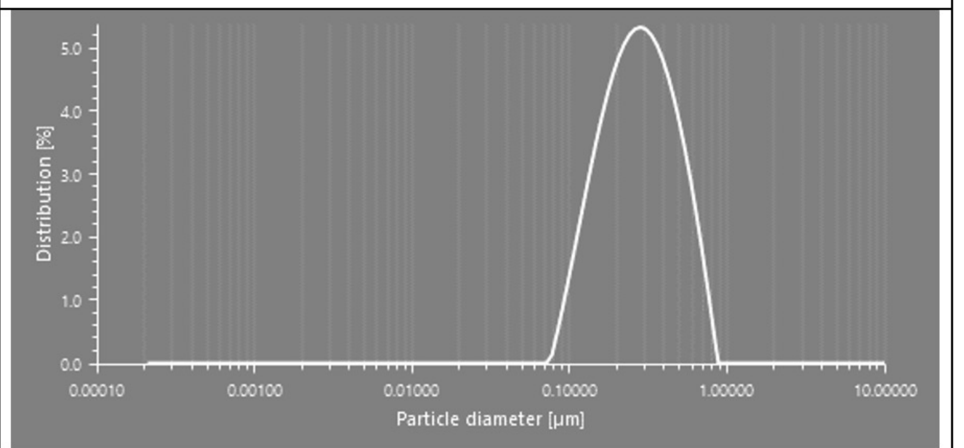

NB3
Hydrodynamic diameter
429.5 nm
PDI: 23.46 %
Peak analysis intensity
799.3 nm 52.31%
173.6 nm 49.69%
Zeta potential: - 20.77 mV

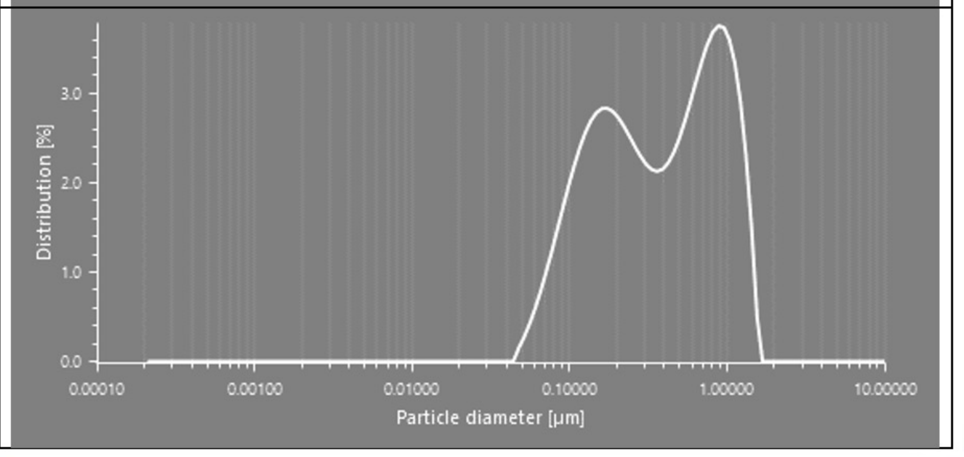

NB4
Hydrodynamic diameter
262.5 nm
PDI: 24.70 %
Peak analysis intensity
392.4 nm 100%
Zeta potential: - 21.20 mV

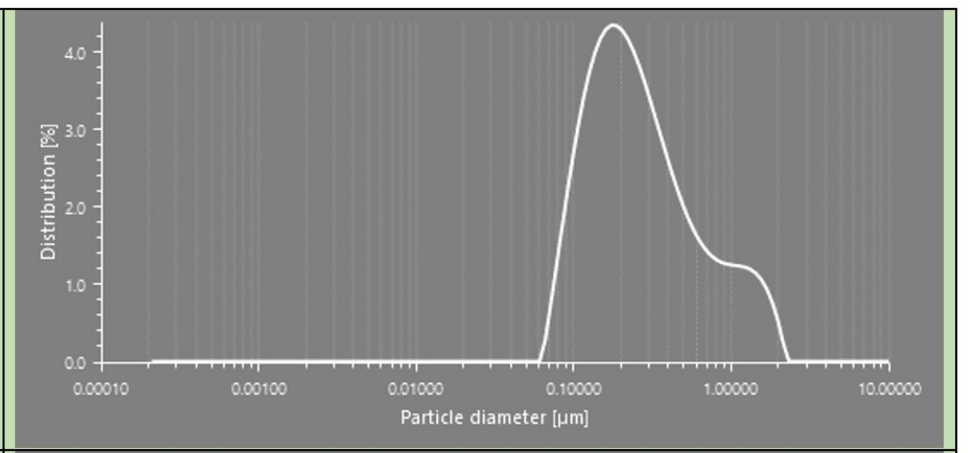

NB5
Hydrodynamic diameter
200.8 nm
PDI: 19.44 %
Peak analysis intensity
244.1 nm 100%
Zeta potential: - 0.026 mV

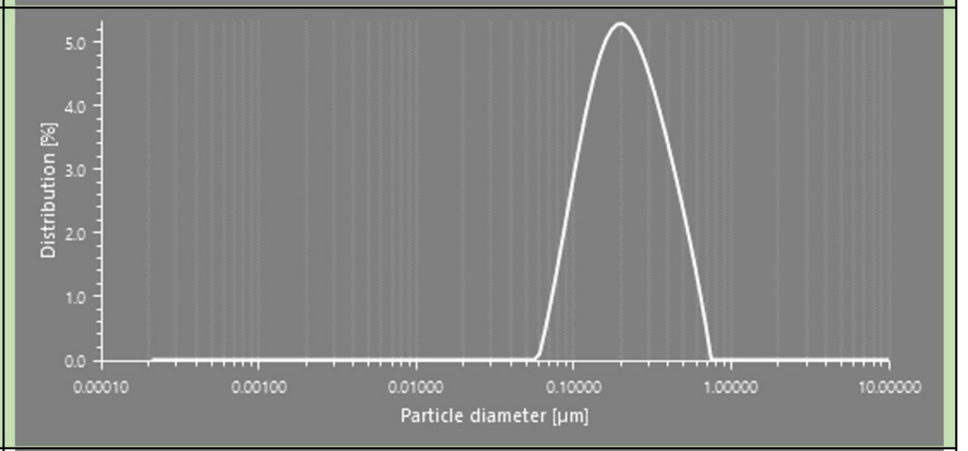

NB6
Hydrodynamic diameter
276.7 nm
PDI: 24.42 %
Peak analysis intensity
305.5 nm 98.10%
29.46 nm 1.90%
Zeta potential: - 12.36 mV

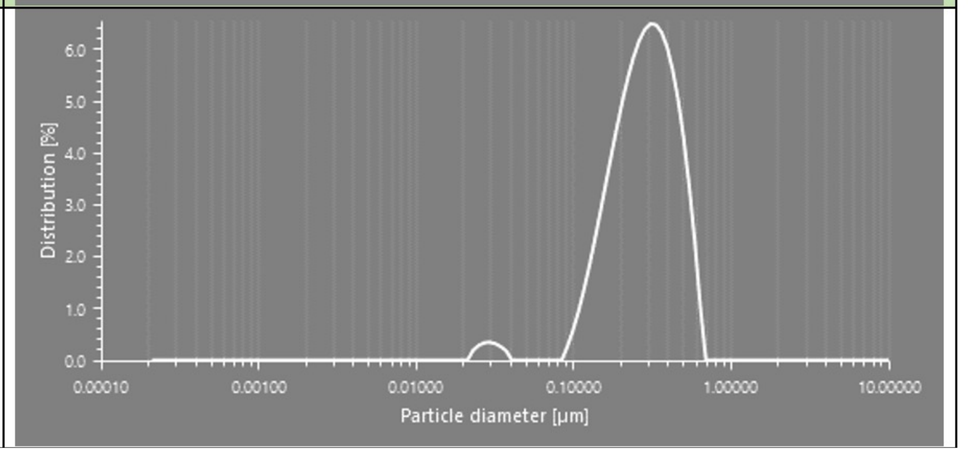

NB7
Hydrodynamic diameter
120.1 nm
PDI: 24.93 %
Peak analysis intensity
140.7 nm 94.93%
13.94 nm 5.07%
Zeta potential: - 23.37 mV

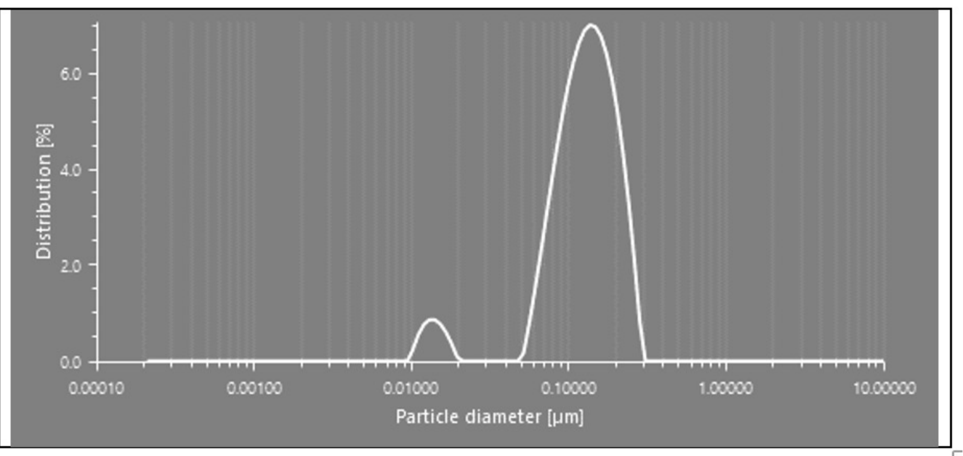

Niosomas NB Span40:Tween 40

NC1
Hydrodynamic diameter
181.0 nm
PDI: 17.79 %
Peak analysis intensity
186.9 nm 100%
Zeta potential: - 33.90 mV

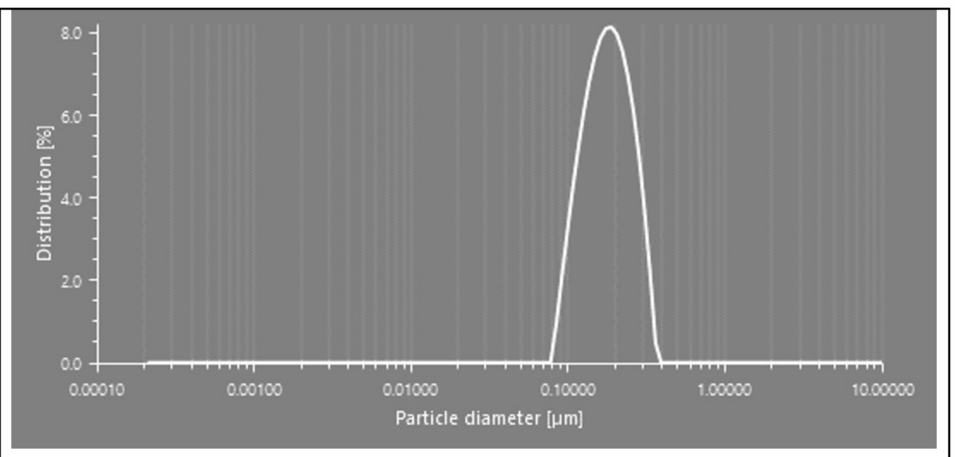

NC2
Hydrodynamic diameter
740.9 nm
PDI: 35.12 %
Peak analysis intensity
919.4 nm 83.01%
121.2 nm 16.99%
Zeta potential: - 39.53 mV

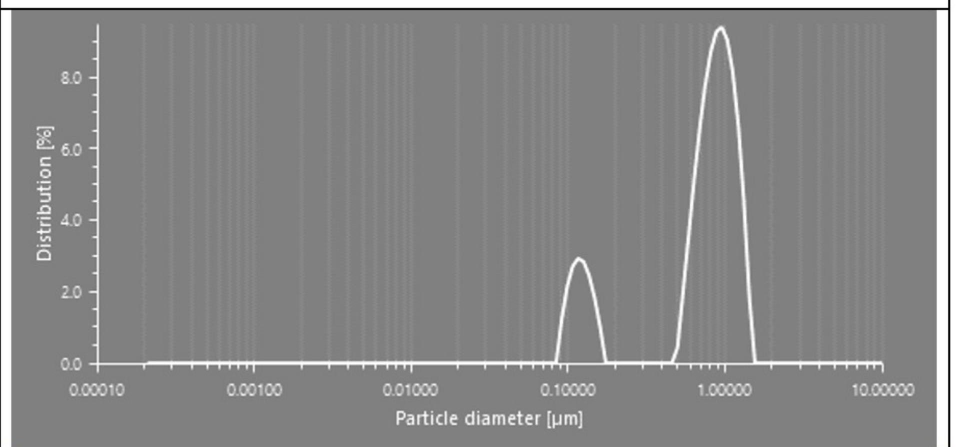

NC3
Hydrodynamic diameter
134.9 nm
PDI: 28.52 %
Peak analysis intensity
211.4 nm 100%
Zeta potential: - 13.2 mV

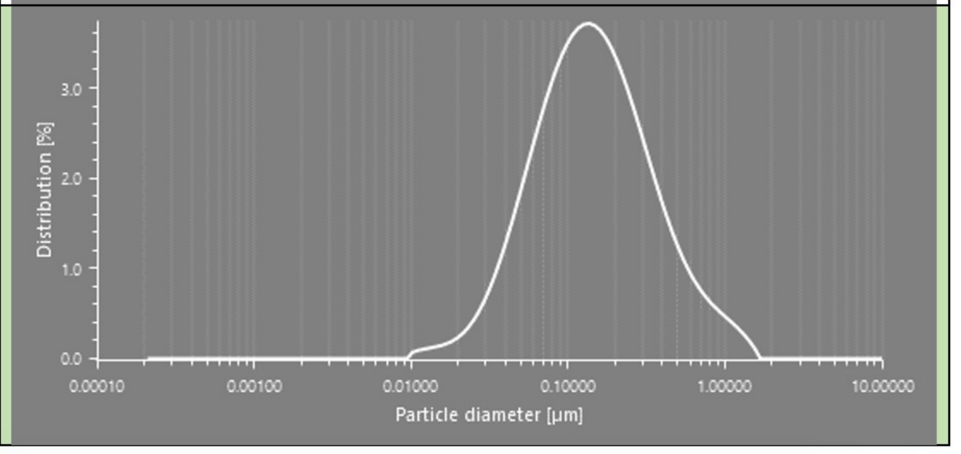

NC4
Hydrodynamic diameter
275.0 nm
PDI: 33.07 %
Peak analysis intensity
654.6 nm 61.61%
57.13 nm 35.19%
12.95 nm 3.21%
Zeta potential: - 27.93 mV

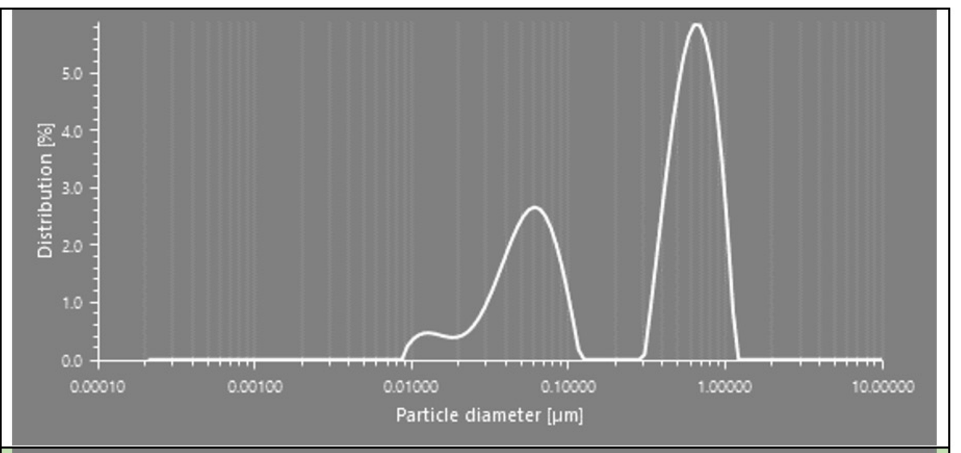

NC5
Hydrodynamic diameter
120.2 nm
PDI: 27.84 %
Peak analysis intensity
95.16 nm 73.91%
621.2 nm 26.09%
Zeta potential: mV

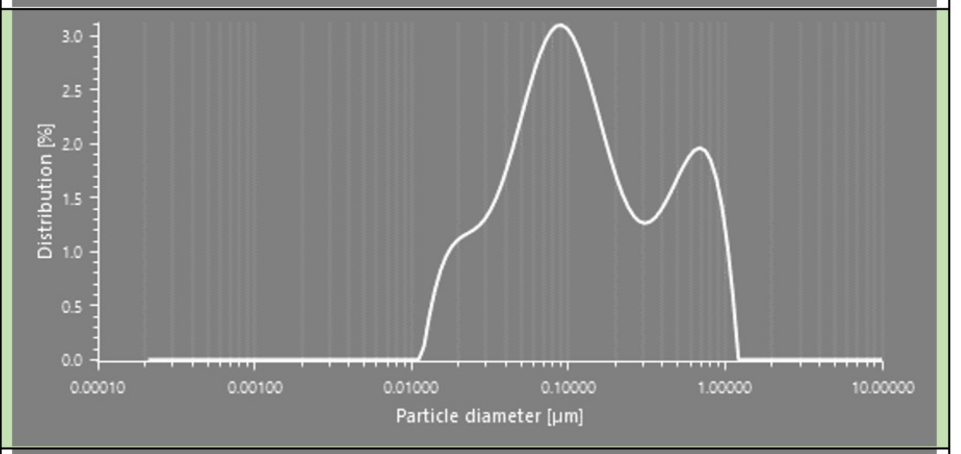

NC6
Hydrodynamic diameter
354.5 nm
PDI: 38.31 %
Peak analysis intensity
1303 nm 46.40%
560.4 nm 28.73%
39.39 nm 24.87%
Zeta potential: mV

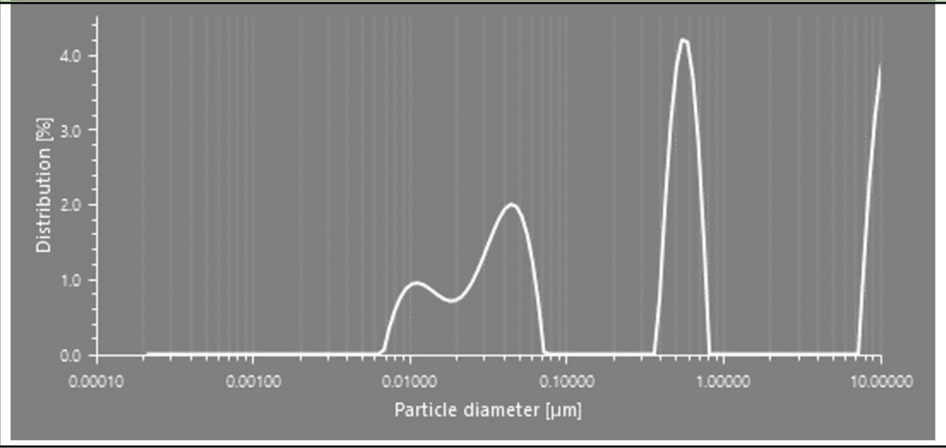

NC7
Hydrodynamic diameter
17.19 nm
PDI: 25.98 %
Peak analysis intensity
13.7 nm 76.90%
727.2 nm 23.10%
Zeta potential: mV

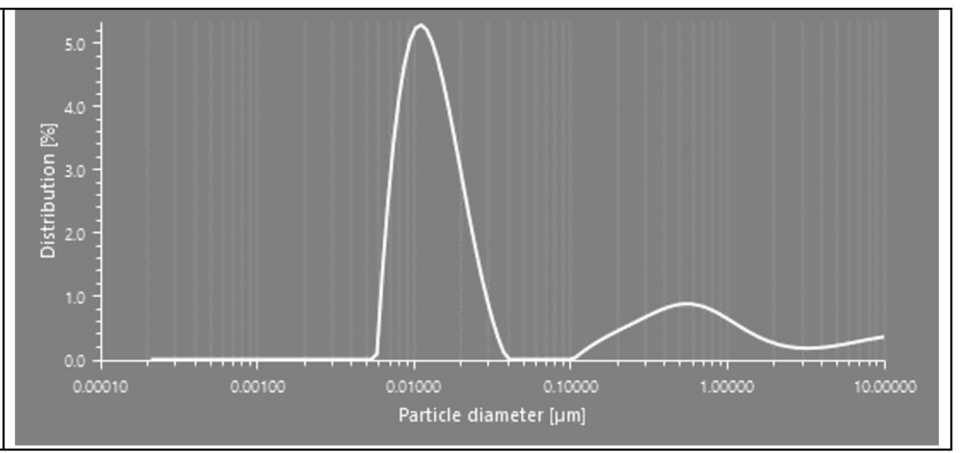

Niosomas ND Span20:Tween 20

ND1 purif
Hydrodynamic diameter
170.4 nm
PDI: 20.84 %
Peak analysis intensity
178.3 nm 100%
Zeta potential: - 44.38 mV

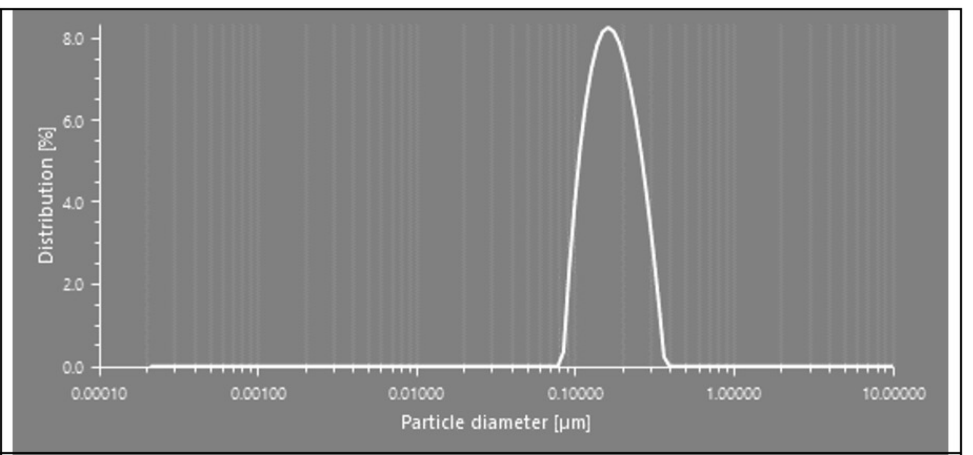

ND2 purif
Hydrodynamic diameter
246.9 nm
PDI: 24.28 %
Peak analysis intensity
263.8 nm 95.13%
29.04 nm 4.87%
Zeta potential: - 25.31 mV

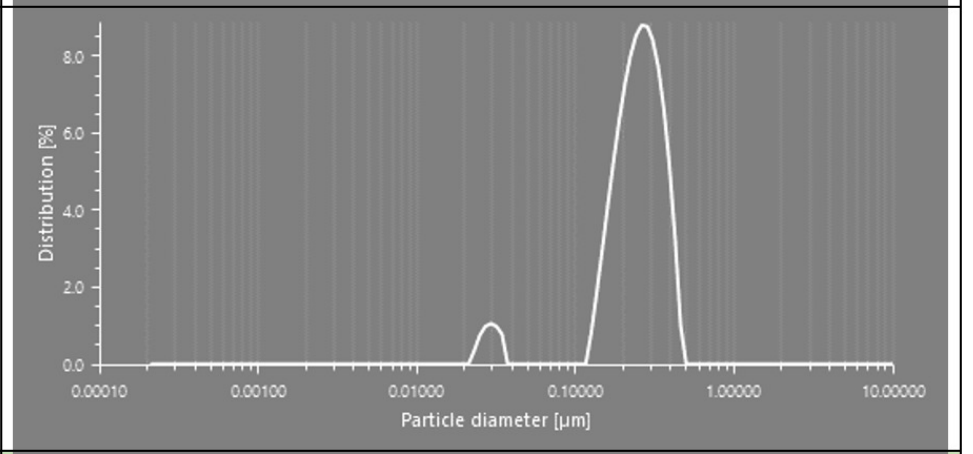

ND3 purif
Hydrodynamic diameter
170.1 nm
PDI: 26.52 %
Peak analysis intensity
218.0 nm 92.48%
36.55 nm 7.52%
Zeta potential: - 17.13 mV

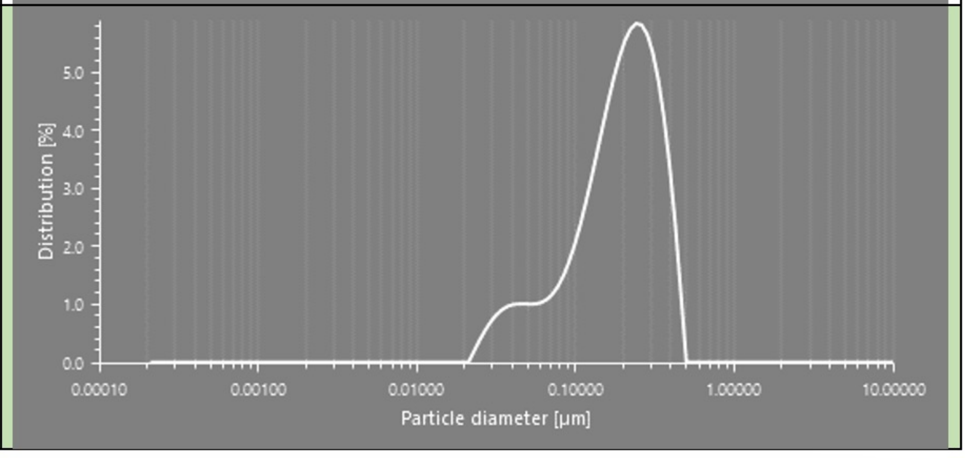

ND4 purif
Hydrodynamic diameter
224.8 nm
PDI: 30.67%
Peak analysis intensity
249.8 nm 84.58%
1455 nm 14.42%
10.33 nm 1.00%
Zeta potential: - 26.00 mV

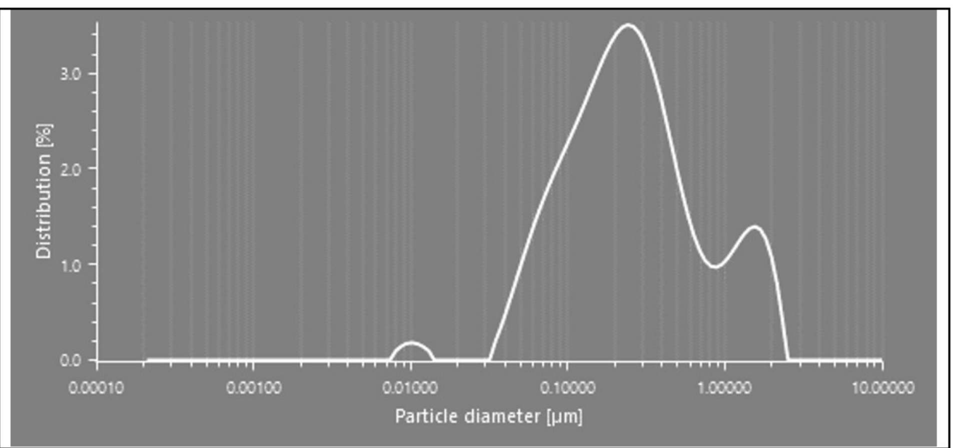

ND5 purif
Hydrodynamic diameter
244.5 nm
PDI: 29.13%
Peak analysis intensity
270.3 nm 92.22%
33.65 nm 7.78%
Zeta potential: - 18.15 mV

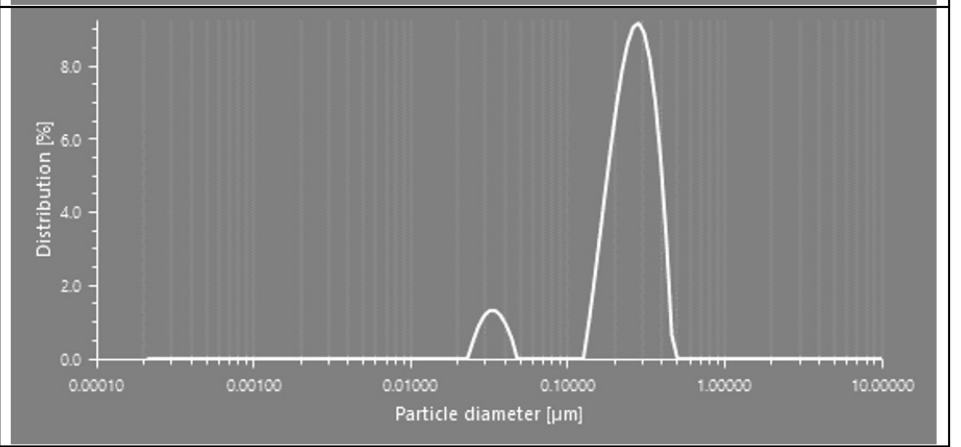

ND6 purif
Hydrodynamic diameter
333.8 nm
PDI: 25.75%
Peak analysis intensity
824.5 nm 42.67%
138.8 nm 55.78%
14.07 nm 1.55%
Zeta potential: - 19.78 mV

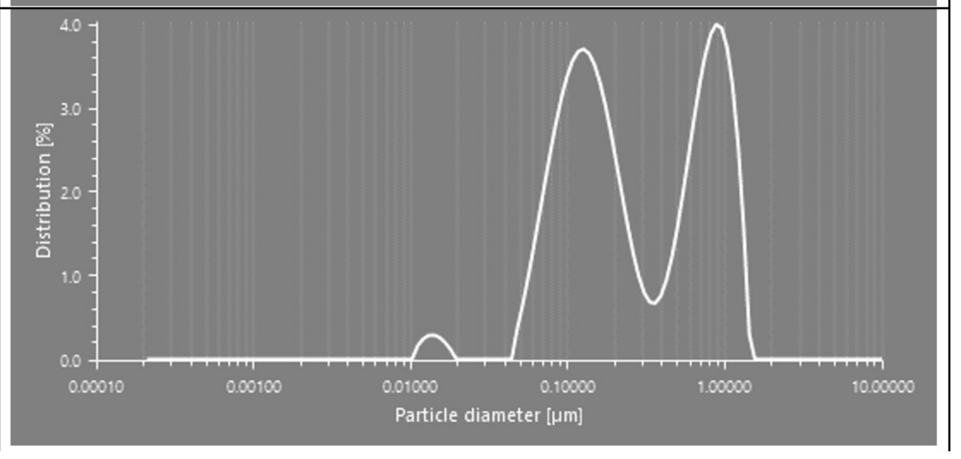

ND7 purif
Hydrodynamic diameter
430.9 nm
PDI: 27.71%
Peak analysis intensity
496.8 nm 93.65%
67.0 nm 6.35%
Zeta potential: + 5.72 mV

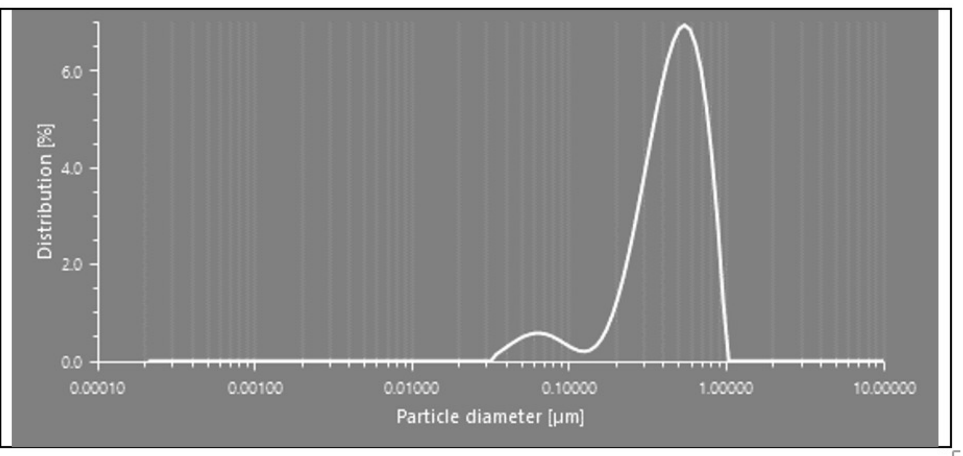

Table S1.

Effect on the particle size of the different surfactants.

Formulation	Molar ratio	D_H Size (nm)	PDI (%)
Brij 58:Chol	3:1	839	27.75
Lubrol PX:Chol	3:1	352	20.54
Span 80:Chol	3:1	270	21.04
Tergitol NP7:Chol	3:1	1490	27.54
Tritón X-100:Chol	3:1	2827	31.41
Tritón X-114:Chol	3:1	2365	20.53
Tween 20:Chol	3:1	751	29.74
Tween 80:Chol	3:1	368	25.80

Chol: cholesterol; D_H: hydrodynamic diameter; PDI: polydispersity index
