# Supplementary material for: AgNP-Containing Niosomes Functionalized with Fucoidan Potentiated the Intracellular Killing of Mycobacterium abscessus in Macrophages
Source: Int J Mol Sci. 2025 Feb 6;26(3):1366. doi: 10.3390/ijms26031366 (PMC11818696; doi:10.3390/ijms26031366)
Supplement: Supplementary file 1 [file ijms-26-01366-s001.zip › ijms-3417443-supplementary-Figure S2.pdf]

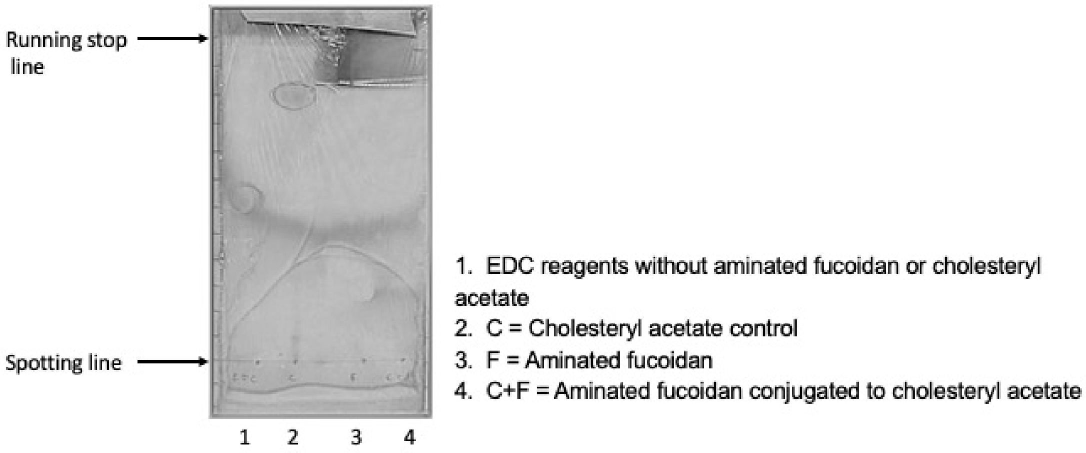


**Figure S2. TLC separation of the different compounds used to conjugate aminated fucoidan and cholesteryl acetate.** The TLC running was performed as described in the Materials and Methods section. Marked spots represent the visible spots observed after charring the TLC plate with vanillin.
